# Inflammatory Markers and Genetic Variants in Gestational Diabetes and Pregnancy Complications: A Cross-Sectional Study

**DOI:** 10.3390/diagnostics15172153

**Published:** 2025-08-26

**Authors:** Jelena Omazić, Andrijana Muller, Mirta Kadivnik, Kristina Kralik, Domagoj Vidosavljević, Jasenka Wagner

**Affiliations:** 1 Department of Laboratory Diagnostic, “Dr. Juraj Njavro” National Memorial Hospital, 32000 Vukovar, Croatia; jelena.omazic@gmail.com; 2 Department of Medical Chemistry, Biochemistry and Clinical Chemistry, Faculty of Medicine, Josip Juraj Strossmayer University, 31000 Osijek, Croatia; 3 Clinic of Obstetrics and Gynecology, University Hospital Center Osijek, 31000 Osijek, Croatiamirta.kadivnik@gmail.com (M.K.); 4 Department of Obstetrics and Gynecology, Faculty of Medicine, Josip Juraj Strossmayer University, 31000 Osijek, Croatia; 5 Department of Medical Statistics and Informatics, Faculty of Medicine, Josip Juraj Strossmayer University, 31000 Osijek, Croatia; 6 Department of Obstetrics and Gynecology, “Dr. Juraj Njavro” National Memorial Hospital, 32000 Vukovar, Croatia; 7 Department of Medical Biology and Genetics, Faculty of Medicine, Josip Juraj Strossmayer University, 31000 Osijek, Croatia

**Keywords:** gestational diabetes, pregnancy, cytokines, inflammation, single nucleotide polymorphism

## Abstract

**Background/Objectives**: Gestational diabetes (GD) is a common pregnancy complication linked to inflammation. Obesity, a major risk factor, is associated with elevated pro-inflammatory markers (TNF-α, IL-6) and reduced anti-inflammatory IL-10 and adiponectin. This study investigated the role of inflammatory factors (IL-6, TNF-α, IL-10, adiponectin) and their genetic variants (rs1800629, rs1800796, rs1800896, rs266729) in a unique four-group study design of pregnant women. **Methods:** We collected venous blood from 162 women in the third trimester of pregnancy. We measured IL-6, IL-10, TNF-α, and adiponectin levels and performed real-time PCR genotyping for the selected SNPs. **Results:** IL-6 levels were significantly higher (*p* < 0.001) in pregnant women with GD and additional complications. The *IL-6* SNP rs1800796 heterozygous CG genotype showed a slightly increased GD risk (OR = 1.41). However, we found no significant associations between GD and *TNF-α* rs1800629 or *IL-10* rs1800896 SNPs. The *AdipoQ rs266729* homozygous CC genotype was linked to increased GD risk (*p* = 0.03 for superdominant model). Importantly, no significant correlations were observed between inflammatory marker levels and gene variants within any study group. **Conclusions:** Our findings suggest a greater inflammatory burden in GD pregnancies with additional complications. While certain *IL-6* and *AdipoQ* variants might contribute to GD risk, the overall weak association between inflammatory markers and gene variants likely reflects the complex polygenic nature of GD, environmental factors, or the study’s sample size.

## 1. Introduction

Gestational diabetes (GD), defined as hyperglycemia first recognized during pregnancy, is the most prevalent metabolic and medical complication in pregnancy, with a growing global incidence [1,2]. Its development is linked to complex etiological and pathophysiological factors, notably involving inflammatory processes. Key inflammatory pathways, such as nuclear factor kappa B (NF-kB) and signal transducer and activator of transcription (STAT3), play crucial roles. NF-kB is central to regulating the inflammatory response and immune system, while STAT3, activated by various pro-inflammatory factors, influences metabolic tissues [3].

Obesity, a significant risk factor for GD, highlights the role of adipose tissue as an endocrine organ secreting adipokines and pro-inflammatory cytokines like tumor necrosis factor α (TNF-α) and interleukin (IL) IL-6. The placenta also contributes to this inflammatory state [4]. TNF-α and IL-6 are particularly implicated, with elevated levels observed in GD patients. TNF-α impairs insulin sensitivity by interfering with insulin signaling, and IL-6 contributes to insulin resistance by increasing hepatic glucose production [5,6]. Conversely, adiponectin, an anti-inflammatory adipokine, is negatively associated with GD and obesity, with lower levels observed in affected pregnancies [4]. Leptin, another adipocyte-derived cytokine, is often elevated in GD, although its precise role is still being elucidated [4,7]. IL-10, an anti-inflammatory cytokine, is crucial for maintaining pregnancy and inhibiting inflammatory responses, but its significance in GD remains to be fully confirmed, with reduced levels potentially linked to adverse pregnancy outcomes [8,9].

Over the past decade, several attempts have been made to assess the relationship between variants in the *TNF-α*, *IL-6*, *IL-10*, and *AdipoQ* genes and the risk of developing GD, but the relationship between these gene variants and the risk of GD remains unclear [10,11,12,13,14,15,16,17,18]. For instance, while some *TNF-α* gene variants show no association with GD, others, like rs1800629, have been identified as risk factors for GD in certain populations [10,11,12]. Similarly, studies have shown that there was no positive association between *IL-6* single nucleotide polymorphism (SNP) rs1800795 which is associated with type 2 diabetes mellitus (T2DM) and type 1 diabetes mellitus (T1DM) [10,13]. In contrast, for SNP rs1800796, the association of allele G with T2DM and GD was found [11,14]. *IL-10* gene polymorphisms present conflicting results regarding their association with GD. No positive association was found between GD and SNPs for rs1800871, rs1800872, while for rs1800896 there are contradictory results, some of which show an association with GD, while others did not [10,11]. In contrast, specific *AdipoQ* SNPs, such as rs2241766, have shown a significant association with GD risk, while rs266729 demonstrates varying effects across different populations [10,15,16,17,18]. Considering the complex interaction of genes and the environment in the development of GD, the genetic variations and their association with GD represent a dynamic area of research with numerous open questions.

This study aims to investigate the complex interplay of inflammatory and anti-inflammatory factors and their genetic predispositions in pregnant women. Specifically, we determined the levels of pro-inflammatory factors (IL-6 and TNF-α) and anti-inflammatory factors (IL-10 and adiponectin). We also analyzed SNPs for key genes, rs1800629 (*TNF-α*), rs1800796 (*IL-6*), rs1800896 (*IL-10*), and rs266729 (*ADIPOQ*), and their relationship with GD. Furthermore, we examined the differences in these inflammatory and anti-inflammatory factor levels and their association with specific gene variants. The aim of this comprehensive approach is to provide deeper insights into the inflammatory mechanisms underlying gestational diabetes and its associated complications.

## 2. Materials and Methods

This study was designed as a cross-sectional investigation. The research was conducted in strict adherence to the Nuremberg Code and the latest revision of the Declaration of Helsinki [19]. All ethical principles were rigorously observed, ensuring the privacy and confidentiality of all participants. Prior to their involvement, all participants received a detailed description of the study and provided written informed consent. The collection and processing of biological material, along with the acquisition of medical documentation, received approval from the Ethics Committee of the Faculty of Medicine University of Osijek (cl: 602-04/23-08/03, number: 2158-61-46-23-142, 26 July 2023), the Ethics Committee of the Osijek University Hospital Centre (number: R1-8243/2023, 14 July 2023) and the Ethics Committee of the “Dr. Juraj Njavro” National Memorial Hospital Vukovar (cl: 510-05/23, registration number: 107-16-23-08-04, 14 July 2023).

A total of 162 pregnant women in their third trimester of pregnancy were included in the study. GD in pregnancy was defined according to the guidelines of the International Association of Diabetes and Pregnancy Study Groups (IADP-SG) [20]. Participants were carefully selected based on specific criteria to ensure the homogeneity and relevance of our study groups. Inclusion criteria mandated that all participants be pregnant women in their third trimester of pregnancy (27th–40th gestational week). The median age of the participants was 31 years, ranging from a minimum of 19 to a maximum of 48 years. We further categorized them into four groups: Group 1: healthy pregnant women with no complications which could have a similar impact on the immune profile as GD (defined by normal glucose, HbA1c, and OGTT results, without GD or other pregnancy pathologies, diabetes, or known autoimmune/metabolic diseases outside of pregnancy); Group 2: healthy pregnant women with complications (who met the normal glucose criteria but had a history of GD, or pre-existing autoimmune diseases, blood pressure disorders, polycystic ovaria syndrome, obesity, or developed these conditions (except GD) during pregnancy); Group 3: pregnant women diagnosed with GD according to IADP-SG guidelines with no other pregnancy complications; and Group 4: pregnant women with GD along with additional complications such as preeclampsia, high blood pressure, or pre-existing/current immune system diseases. Conversely, exclusion criteria were strictly applied to maintain the integrity of the study. Women were excluded if they did not provide informed consent, had a pre-existing diagnosis of diabetes outside of pregnancy, or had any other known metabolic diseases outside of pregnancy. Multiple pregnancies were also excluded from the study.

For each pregnant woman, one tube of peripheral venous blood was collected into a BD Vacutainer (Beckton Dickinson, Franklin Lakes, NJ, USA) containing K3EDTA anticoagulant, and one serum tube of peripheral venous blood was collected into a BD Vacutainer. Analyses were performed on fresh whole blood and serum samples. Serum was prepared by centrifuging the serum tube at 3000× *g* for 10 min at room temperature. IL-6 and C-reactive protein (CRP) analysis were performed on a cobasPro automated analyzer (Roche Diagnostic, Basel, Switzerland). IL-10 and TNF-α analyses were conducted using chemiluminescent immunoassay methods on an Immulite 2000 analyzer (Siemens Healthineers, Erlangen, Germany) with sensitivity limitation of both tests of 5 pg/mL Adiponectin levels were determined in serum using an ELISA method (BioVendor R&D, Brno, Czech Republic). For the adiponectin ELISA, commercial standards, two levels of quality controls, and participant samples were incubated on an ELISA plate coated with recombinant human adiponectin. Polyclonal anti-adiponectin antibodies conjugated with horseradish peroxidase (HRP) were then added. After washing, tetramethylbenzidine substrate was added to the adiponectin-HRP conjugate complex, and the reaction was stopped after 15 min with an acidic STOP solution. Absorbance was measured spectrophotometrically at 450 nm and 630 nm. Absorbance was inversely proportional to the adiponectin concentration in the samples. A standard curve was generated using known concentrations and corresponding absorbances of commercial standards in Microsoft Excel, from which participant sample values were extrapolated.

Genomic Deoxyribonucleic Acid (DNA) was isolated using a commercial kit according to the manufacturer’s protocol (QIAamp DNA Blood Midi Kit, Qiagen, Hilden, Germany). DNA isolation commenced with the incubation of a mixture containing 200 μL of participant whole blood, 200 μL of lysis buffer, and 20 μL of proteinase K. The mixture was incubated for 10 min at 56 °C. The addition of 1000 μL of 100% ethanol led to DNA precipitation, which was then transferred to a silica membrane within a spin column. The mixture was centrifuged for 1 min at 6000× *g*, allowing the DNA to bind to the membrane. The isolated DNA was further purified by adding 500 μL of AW1 buffer and centrifuging at 6000× *g* for 1 min. The QIAamp Mini spin column was then transferred to a new tube, 500 μL of AW2 buffer was added, and it was centrifuged at 20,000× *g* for 3 min. The QIAamp Mini spin column was then transferred to a new tube, and 100 μL of AE buffer was added. The mixture was incubated at room temperature for 1 min and then centrifuged at 6000× *g* for 1 min. Isolated DNA was stored at −20 °C in AE buffer until analysis. The concentration of isolated DNA was determined fluorometrically using a Qubit instrument (Thermo Fisher Scientific, Waltham, MA, USA). Following DNA isolation and quantification, genotyping of single nucleotide polymorphisms was performed using real-time Polymerase Chain Reaction (PCR). The 7500 Real-Time PCR System (Applied Biosystems, Foster City, CA, USA) was used for genotyping, configured with a genotyping program utilizing TaqMan probes (TaqMan SNP Genotyping Assays, Life Technologies, Carlsbad, CA, USA). Genotypes were determined for the following SNPs: rs1800629 (*TNF-α*), rs1800796 (*IL-6*), rs1800896 (*IL-10*), and rs266729 (*AdipoQ*). Specifically, SNP rs1800629 is located in the intronic region of the *TNF-α* gene at chr6:31575254 (GRCh38.p14), commonly featuring a G allele which can be substituted by an A allele. The *IL-6* gene is on chromosome 7, with SNV rs1800796 at chr7:22726627 (GRCh38.p14), exhibiting G > A/G > C allele variations. The *IL-10* gene is on chromosome 1, with SNP rs1800896 at chr1:206773552 (GRCh38.p14), showing T > C allele variants. Lastly, within the adiponectin gene, rs266729 is at chr3:186841685 (GRCh38.p14), where C > A/C > G/C > T allele substitutions can occur. TaqMan™ Universal PCR Master Mix (Thermo Fisher Scientific, Foster City, CA, USA) and TaqMan™ SNP Genotyping Assays (Thermo Fisher Scientific, Foster City, CA, USA) were used for the PCR reaction. The total reaction volume per well was 25 μL, containing 1 μL of isolated DNA.

Categorical data are presented as absolute and relative frequencies. Continuous data are described by median and interquartile range limits. The Shapiro–Wilk test was used to assess the normality of the distribution of continuous variables. For more than two independent groups, differences in continuous variables that did not follow a normal distribution were tested by the Kruskal–Wallis test (with Conover post hoc analysis). Bonferroni correction was performed for all multiple testing. The statistical power of the allelic association test for the polymorphism was calculated using the Genetic Power Calculator (http://zzz.bwh.harvard.edu/gpc/cc2.html (accessed on 10 August 2024)). Hardy–Weinberg equilibrium of genotype frequencies was tested by the chi-square test (df = 1) using the Hardy–Weinberg Equilibrium Calculator (https://wpcalc.com/en/equilibrium-hardy-weinberg/ (accessed on 9 August 2024)). All *p*-values were two-sided, and the significance level was set at 0.05. For statistical analysis, we used the statistical software MedCalc version 14.12.0 (MedCalc Software bvba, Ostend, Belgium; http://www.medcalc.org; 2014, (accessed on 10 August 2024)) and SPSS Statistics 23 (IBM Corp. Released 2015. IBM SPSS Statistics for Windows, version 23.0. Armonk, NY, USA: IBM Corp.).

## 3. Results

Statistical analysis of the data showed that there was a statistically significant difference in IL-6 concentrations at the *p* < 0.05 level between the group of pregnant women with gestational diabetes and complications (Group 4) compared to the remaining three groups of subjects (Table 1).

Table 2, Table 3, Table 4 and Table 5 show the frequency and distribution of genotypes and alleles of the analyzed SNPs in the control groups and in the groups of subjects with GD.

Our investigation showed that there were no significant differences in the values of IL-6, IL-10, TNF-α, and adiponectin with respect to the genotypes of the analyzed SNPs in each group of subjects (Table 6).

## 4. Discussion

Our findings indicate that the highest IL-6 levels were observed in pregnant women with GD complicated by additional conditions, demonstrating a statistically significant difference compared to the other three study groups. Given that IL-6 is a well-established marker of inflammation, these results suggest that pregnancies concurrently affected by GD and other complications represent a heightened inflammatory state compared to pregnancies with GD alone or those complicated by other comorbidities such as hypertension or thyroid dysfunction. This observation is particularly relevant when considering the broader literature. A review by Amirian et al. [21] analyzed 24 qualified studies, with 16 demonstrating a statistically significant association between IL-6 and GD, while 8 studies found no such association. In light of our present findings, where the highest IL-6 levels were seen in the group with GD and additional complications, it is suggested that IL-6 elevation may not be exclusively linked to GD itself, but rather to the combined inflammatory burden of GD alongside other complicating factors. We also investigated the association between SNP rs1800769 in the *IL-6* gene and the risk of GD by analyzing the frequencies of GG, CG, and CC genotypes in a control group of pregnant women and a group of pregnant women with GD, using codominant, dominant, recessive, and superdominant inheritance models. We chose to analyze the variants under codominant, dominant, recessive, and superdominant inheritance models because these approaches allow for a comprehensive assessment of potential genotype–phenotype associations. Genetic effects can manifest differently depending on whether one or both alleles influence the trait. The codominant model provides an unbiased view of all three genotypes, while dominant and recessive models test for situations in which one allele is sufficient to drive the effect. The superdominant model is useful for detecting potential heterozygote advantage or disadvantage. Including all four models ensures that we do not miss possible biological patterns of association that might otherwise remain undetected [22,23]. Genotype frequencies for IL-6 in both groups conformed to Hardy–Weinberg equilibrium, indicating random mating and no significant deviations from expected frequencies. CRP levels were also measured in all pregnant women. There was a statistically significant difference between Group 1 and the other three groups; specifically, pregnant women without gestational diabetes or other complications had the lowest CRP values.

The odds ratio (OR) represents the relative risk of developing GD for carriers of a specific genotype compared to carriers of the reference genotype. For the heterozygous CG genotype at SNP rs1800796, the OR was 1.41, suggesting a slightly increased risk for GD in carriers of this genotype. In the dominant and superdominant models, the ORs for the combined CG and CC genotypes were 1.54 and 1.39, respectively, also indicating a marginally elevated risk for GD. The OR for the CC genotype in the recessive model could not be calculated due to its low frequency. Although the odds ratio for the rs1800796 SNP suggests a slightly increased risk, it is important to note that the *p*-values for both the codominant and dominant models are not statistically significant. This indicates that the observed association could be due to chance, and we cannot conclude a definitive link between this specific SNP and the outcome in our study population. This finding highlights the need for larger studies to confirm any potential association. Our findings align with previous studies that reported an association of the G allele in polymorphism rs1800796 with both T2DM and GD [11,14]. In contrast, no statistically significant differences were found in the distribution of genotypes and alleles for SNP rs1800629 (*TNF-α*) and rs1800896 (*IL-10*) between healthy pregnant women and those with GD, regardless of the inheritance model (codominant, dominant, recessive, superdominant). The odds ratio values for these SNPs were close to 1, indicating no substantial increased likelihood of developing GD in carriers of specific genotypes. Small deviations from Hardy–Weinberg equilibrium in both groups suggest random mating and the absence of significant departures from expected genotype frequencies. These results contradict some existing knowledge regarding these polymorphisms. Specifically, while previous research linked the G allele of rs1800796 to T2DM and GD [11,14], our study did not confirm this association for GD. Similarly, polymorphism rs1800896 was previously associated with an increased risk for T2DM [24], but our investigation found no such link with GD.

Analysis of polymorphism rs266729 in the *AdipoQ* gene suggests a potential influence of adiponectin genotype on GD development. The homozygous CC genotype was associated with an increased risk of GD compared to the heterozygous CG genotype. This association reached statistical significance in the dominant model (*p* = 0.08). The odds ratio for the homozygous CC genotype was greater than 1 relative to the heterozygous CG genotype, indicating an increased risk of GD for CC genotype carriers. These findings, however, contradict previous results where the G allele of rs266729 in the *AdipoQ* gene was shown to increase the risk of GD [15,16,17,18]. Genotype frequencies for adiponectin in both the control and GD groups conformed to Hardy–Weinberg equilibrium. This discrepancy with prior research may be attributed to several factors. First, genetic associations can vary across different ethnic and geographic populations, and it is possible that the biological effect of the CC genotype differs in our specific study population. Second, GD is a complex multifactorial disease, and the effect of a single SNP can be influenced by interactions with other genes and environmental factors that may not have been accounted for in our study. Finally, a significant limitation of our study is the small sample size, which may have prevented us from achieving statistical significance for some of our findings. However, we did observe notable, non-significant trends that warrant further investigation. For example, both the rs1800796 and rs266729 SNPs showed trends toward an increased risk of GD. These observations, particularly those that contradict some prior research, highlight the need for larger, well-powered studies to definitively clarify the role of these SNPs, and also other SNPs, in the development of gestational diabetes.

To our knowledge, this is the first study to investigate the relationship between IL-6, IL-10, TNF-α, and adiponectin levels, and the genetic variants rs1800629, rs1800796, rs1800896, and rs266729. Our analysis revealed no significant differences in the levels of these inflammatory and anti-inflammatory markers across the analyzed genotypes in either the control group or the GD group, nor within individual subgroups of participants. The lack of significant associations might be attributed to the polygenic nature of complex traits like inflammatory marker levels. It is plausible that other genes, or combinations of genes, are responsible for the observed variations in these pro-inflammatory and anti-inflammatory factors. Beyond genetic predispositions, environmental factors such as Body Mass Index (BMI), diet, physical activity, and stress also significantly influence inflammatory and metabolic markers [25]. These environmental influences could have potentially masked or modified any subtle genetic effects in our study. Furthermore, the relatively small sample size of 162 pregnant women in this study might have limited our ability to detect smaller, yet statistically significant, differences between the investigated groups. Therefore, we cannot rule out the possibility that a larger cohort study could reveal statistically significant associations that were not evident in our current investigation.

The findings of this study significantly advance our understanding of the pathophysiology of GD. This is the first investigation to utilize a four-group subject design, meticulously categorizing participants as healthy pregnant women, healthy pregnant women with immunological complications, pregnant women with GD, and pregnant women with both GD and immunological complications. Adherence to strict inclusion and exclusion criteria ensured the homogeneity of our cohorts, which were well-matched across critical parameters including age, gestational week, immunological profile, and previous medical history. A notable strength of our methodology was the recruitment of all pregnant women from the same geographical region, coupled with a uniform number of subjects across all groups.

A primary limitation of this study was the relatively small sample size, which may have constrained our ability to detect more subtle associations. Furthermore, the determination of IL-10 levels via an immunochemical method presented a challenge. All subjects exhibited IL-10 values below 5 pg/mL, a concentration at which immunochemical methods inherently possess limited sensitivity. This restricted the scope of our statistical analysis. It is important to note that the automated IL-10 determination method employed in this study is a recent market introduction. We anticipate that future advancements in this methodology will improve sensitivity limits, enable more precise IL-10 quantification and facilitate comprehensive correlational analyses with IL-10 gene variants. Also, a limitation of our study is the low odds ratio values found for several characteristics. These values suggest a weak association, and we caution that our findings should be interpreted with care. Future studies should aim to validate these relationships using larger, more diverse populations to establish stronger evidence.

Future research could build upon these findings by exploring additional gene variants for the pro-inflammatory and anti-inflammatory factors examined in this study, alongside an expansion of subject cohorts. Furthermore, it would be valuable to subcategorize pregnant women with complications based on the specific type of complication. This would allow for a more nuanced investigation into whether different complications exert equivalent effects on the pregnant woman’s immune profile, ultimately leading to a more refined understanding of GD pathophysiology.

## Figures and Tables

**Table 1:** Differences in cytokines and inflammatory markers between groups.

	Median (Interquartile Range)				*p* *
	Group 1	Group 2	Group 3	Group 4	
	(1)	(2)	(3)	(4)	
IL-6	2.4 (1.85–3.4)	2.6 (1.9–3.9)	2.3 (1.5–3.2)	3.25 (2.53–4.4)	**<0.001**
IL-10	0 (0–0)	0 (0–0)	0 (0–0)	0 (0–0)	>0.99
TNF-α	4.29 (0–5.5)	0 (0–4.2)	4.2 (0–5.2)	4.47 (0–5.6)	0.08
adiponectin	16 (3.2–33.1)	7.65 (4.53–14.5)	17.6 (6.15–30.1)	17.2 (6.53–22.3)	0.09
C-reactive protein	2.7 (1.43–4.2)	4 (2.75–7.4)	4.35 (2.5–8)	6.35 (2.38–8.9)	**<0.001**

IL—interleukin, TNF—tumor necrosis factor; * Kruskal–Wallis test (post hoc Conover). Bold denotes statistical significance.

**Table 2:** Frequency of genotypes and alleles of rs1800796 (*IL-6*) between the group of subjects with GD and healthy subjects.

		Genotype [*n* (%)]		Odds Ratio (95% Confidence Interval)	*p*
		Control	GD		
codominant	GG	72 (90)	70 (85)	1.0	0.39 *
	CG	8 (10)	11 (14)	1.41 (0.54–3.72)	
	CC	0	1 (1)	-	
allele	G	152 (95)	151 (92)	1.64 (0.66–4.06)	0.29 ^†^
	C	8 (5)	13 (8)		
dominant	GG	72 (90)	70 (85)	1.0	0.37 *
	CG-CC	8 (10)	12 (15)	1.54 (0.59–4.0)	
recessive	GG-CG	80 (100)	81 (99)	1.0	0.24 *
	CC	0	1 (1)	-	
superdominant	GG-CC	72 (90)	71 (87)	1.0	0.50 *
	CG	8 (10)	11 (13)	1.39 (0.53–3.67)	

Hardy–Weinberg equilibrium—control = 1.0; Hardy–Weinberg equilibrium—GD = 0.4; * χ^2^ test; ^†^ Fisher exact test; GD—gestational diabetes.

**Table 3:** Frequency of genotypes and alleles of rs1800896 (*IL-10*) between the group of subjects with GD and healthy subjects.

		Genotype [*n* (%)]		Odds Ratio (95% Confidence Interval)	*p* *
		Control	GD		
codominant	TT	30 (38)	30 (36)	1.0	0.95
	TC	35(43)	35 (43)	1.0 (0.50–1.99)	
	CC	15 (19)	17 (21)	1.13 (0.48–2.68)	
allele	T	95 (59)	95 (58)	1.06 (0.68–1.65)	0.79
	C	65 (41)	69 (42)		
dominant	TT	30 (37)	30 (37)	1.0	0.90
	TC-CC	50 (63)	52 (63)	1.04 (0.55–1.97)	
recessive	TT-TC	65 (81)	65 (79)	1.0	0.89
	CC	15 (19)	17 (21)	1.13 (0.52–2.46)	
superdominant	TT-CC	45 (56)	47 (57)	1.0	0.89
	TC	35 (44)	35 (43)	0.96 (0.51–1.78)	

Hardy–Weinberg equilibrium—control = 0.49; Hardy–Weinberg equilibrium—GD = 0.26; * χ^2^ test; GD—gestational diabetes.

**Table 4:** Frequency of genotypes and alleles of rs1800629 (*TNF-α*) between the group of subjects with GD and healthy subjects.

		Genotype [*n* (%)]		Odds Ratio (95% Confidence Interval)	*p*
		Control	GD		
codominant	GG	57 (71)	63 (77)	1.0	0.70 *
	AG	20 (25)	16 (20)	0.72 (0.34–1.53)	
	AA	3 (4)	3 (4)	0.90 (0.18–4.66)	
allele	G	134 (84)	142 (87)	0.79 (0.43–1.48)	0.47 ^†^
	A	26 (16)	22 (13)		
dominant	GG	57 (71)	63 (77)	1.0	0.70 *
	AA-AA	3 (4)	3 (4)	0.90 (0.18–4.66)	
recessive	GG-AG	77 (96)	79 (96)	1.0	0.98 *
	AA	3 (4)	3 (4)	0.97 (0.19–4.98)	
superdominant	GG-AA	60 (75)	66 (81)	1.0	0.40 *
	AG	20 (25)	16 (19)	0.73 (0.35–1.53)	

Hardy–Weinberg equilibrium–control = 0.42; Hardy–Weinberg equilibrium–GD = 0.15; * χ^2^ test; ^†^ Fisher exact test; GD—gestational diabetes.

**Table 5:** Frequency of genotypes and alleles of rs266729 (*AdipoQ*) between the group of subjects with GD and healthy subjects.

		Genotype [*n* (%)]		Odds Ratio (95% Confidence Interval)	*p* *
		Control	GD		
codominant	CC	31 (39)	43 (53)	1.0	0.09
	CG	44 (55)	31 (38)	0.51 (0.26–0.97)	
	GG	5 (6)	8 (9)	1.15 (0.34–3.86)	
allele	C	106 (66)	117 (47)	0.79 (0.49–1.26)	0.32
	G	54 (34)	47 (29)		
dominant	CC	31 (39)	43 (53)	1.0	0.08
	CG-GG	49 (61)	39 (47)	0.57 (0.31–1.07)	
recessive	CC-CG	75 (94)	74 (90)	1.0	0.41
	GG	5 (6)	8 (10)	1.62 (0.51–5.19)	
superdominant	CC-GG	36 (45)	51 (62)	1.0	**0.03**
	CG	44 (55)	31 (38)	0.50 (0.27–0.93)	

Hardy–Weinberg equilibrium—control = 0.04; Hardy–Weinberg equilibrium—GD = 0.59; * χ^2^ test; GD—gestational diabetes. Bold denotes statistical significance.

**Table 6:** Differences in IL-6, IL-10, TNF-α, and adiponectin values with respect to the genotypes of the analyzed SNPs in each group of subjects.

		Median (Interquartile Range)			*p* *
	rs1800796 (*IL-6*)	CC	CG	GG	
IL-6	Group 1	-	2.4 (2.1–3.8)	2.4 (1.8–3.4)	0.80
	Group 2	-	1.5 (*n* = 1)	2.6 (1.9–3.9)	0.13
	Group 3	-	2.0 (1.5–4.2)	2.3 (1.6–3.3)	0.60
	Group 4	6.5 (*n* = 1)	4.1 (2.9–5.7)	3.1 (2.5–4.2)	0.25
	rs1800896 (*IL-10*)	CC	TC	TT	
IL-10	Group 1	-	-	-	-
	Group 2	-	-	-	-
	Group 3	-	-	-	-
	Group 4	-	-	-	-
	rs1800629 (*TNF-α*)	AA	AG	GG	
TNF-α	Group 1	-	4.3 (0–6.2)	2.2 (0–4.9)	0.62
	Group 2	0 (0–9.7)	0 (0–1.0)	0 (0–4.6)	0.79
	Group 3	2.1 (0–5.5)	0 (0–5)	4.5 (0–5.4)	0.49
	Group 4	0 (*n* = 1)	0 (0–5.2)	4.8 (0–5.8)	0.19
	rs266729 (*AdipoQ*)	CC	CG	GG	
adiponectin	Group 1	12.8 (3.8–27.7)	19.4 (2.3–44.6)	21.9 (15.2–18.7)	0.82
	Group 2	7.6 (4.7–21.3)	8.4 (4.6–14.5)	4.6 (3.2–25.0)	0.88
	Group 3	18.0 (5.4–21.3)	15.1 (8.3–30.4)	13.8 (3.9–50.5)	0.99
	Group 4	17.2 (5.3–29.6)	15.4 (7.9–21.8)	21.4 (19.1–24.7)	0.54

* Kruskal–Wallis test; IL—interleukin, TNF—tumor necrosis factor.

## Data Availability

The data that support the findings of this study are available as a Supplementary Material (Table S1).

## Supplementary Materials

The following supporting information can be downloaded at: https://www.mdpi.com/article/10.3390/diagnostics15172153/s1, Table S1. (General data, Lifestyle, Medical history, Gynecological and obstetrical anamnesis, OGTT, Actual pregnancy data, Cytokines levels, Cytokines genotypes).

## Abbreviations

The following abbreviations are used in this manuscript:

GD	gestational diabetes
NF-kB	nuclear factor kappa B
STAT3	signal transducer and activator of transcription (STAT3)
TNF-α	tumor necrosis factor α
IL	interleukin
T2DM	type 2 diabetes mellitus
T1DM	type 1 diabetes mellitus
SNP	single nucleotide polymorphism
IADP-SG	International Association of Diabetes and Pregnancy Study Groups
HRP	horseradish peroxidase
DNA	deoxyribonucleic acid
PCR	polymerase chain reaction
